# A nomogram prediction model for plastic bronchitis in children with refractory *Mycoplasma pneumoniae* pneumonia

**DOI:** 10.3389/fped.2026.1667816

**Published:** 2026-03-31

**Authors:** Hongcai He, Ziyang Kuang, Zhongfa Zhang, Guolin Rao, Chen Xiong, Liqun Lu, Changqiang Yang, Yue Song

**Affiliations:** 1 Department of Pediatrics, The Third Affiliated Hospital of Chengdu Medical College, People's Hospital of Pi County, Chengdu, Sichuan, China; 2 Department of Pediatrics, The First Affiliated Hospital of Chengdu Medical College, Chengdu, Sichuan, China; 3 Department of Cardiology, The First Affiliated Hospital of Chengdu Medical College, Chengdu, Sichuan, China

**Keywords:** children, *Mycoplasma pneumoniae* pneumonia, nomogram model, plastic bronchitis, predictive factors

## Abstract

**Objective:**

To determine the risk factors for plastic bronchitis (PB) in children diagnosed with *Mycoplasma Pneumoniae* Pneumonia (RMPP) and facilitate early intervention.

**Methods:**

A retrospective study of 205 hospitalized children diagnosed with RMPP in two tertiary hospitals was conducted from January 2023 to May 2025. The children were divided into the PB group and non-PB group. Clinical characteristics, laboratory indices, pulmonary imaging findings, and treatment approaches were compared between the two groups. A nomogram model was established based on logistic regression to assess the risk of PB in children infected with RMPP.

**Results:**

A total of 52 patients (25.4%) were included in the PB group. The nomogram model constructed in this study indicated that three risk factors- C-reactive protein (CRP) > 20 mg/L, pleural effusion, and high Lactate Dehydrogenase (LDH) levels- could be used for the early identification of PB in children with RMPP. The area under the receiver operating characteristic curve of the prediction model was 0.783 (95%CI: 0.71–0.86). The Hosmer–Lemeshow goodness-of-fit test demonstrated the good calibration of the nomogram [(*P* = 0.408, R^2^ = 8.269)]. Decision curve analysis showed that the model had clinical value.

**Conclusions:**

Early identification of these risk factors (CRP > 20 mg/L, pleural effusion, and elevated LDH) may facilitate timely bronchoscopic examination in children with RMPP at high risk of PB, potentially contributing to improved clinical management.

## Introduction

*Mycoplasma pneumoniae* (MP) is a major pathogen causing community-acquired pneumonia in children, especially in school-age children, with an incidence rate of 28%–50% (1–4). Refractory *Mycoplasma pneumoniae* pneumonia (RMPP) is defined as persistent fever and radiographic progression despite adequate macrolide treatment. This condition has received increasing attention and poses significant clinical challenges (3–5). RMPP is associated with several severe complications, including pleural effusion, pulmonary embolism, pulmonary necrosis, and particularly plastic bronchitis(PB) (5–7). PB, a rare and severe pulmonary disease in children, is primarily diagnosed through bronchoscopy. It involves the formation of obstructive mucous casts within the airways, leading to symptoms such as atelectasis and dyspnea, and may cause severe complications such as respiratory failure or death (1–3). The emergence of RMPP has further complicated treatment and is increasingly linked to the development of PB (7–9). The pathogenesis of PB is thought to involve intense inflammation, airway injury, impaired mucociliary clearance, and viscous secretions (10, 11). With the increasing application of bronchoscopy in severe pneumonia, more and more cases of RMPP complicated with PB have been identified (7, 9).

Early identification of PB in children with RMPP is crucial for timely bronchoscopic intervention to remove the casts, thereby improving outcomes and reducing adverse prognoses (6, 7). However, predicting the occurrence of PB in RMPP patients remains challenging. This study aimed to identify the risk factors for PB in RMPP and develop a practical predictive nomogram based on clinical characteristics to facilitate early diagnosis and intervention.

## Method

### Study subjects

**Figure 1:**
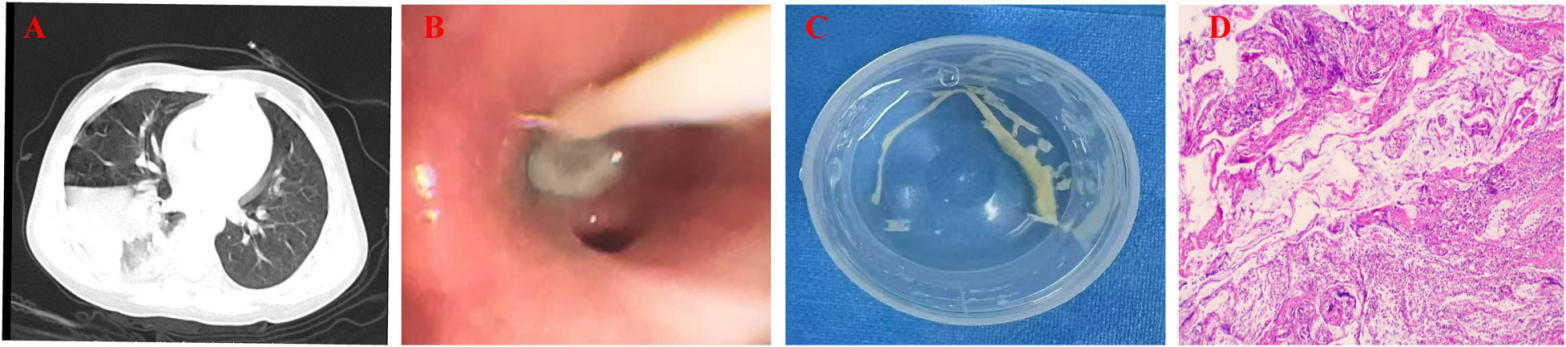
The discovery of an 8 -year-old boy with RMPP. **(A)** The chest CT scan upon admission. The chest CT showed large areas of consolidation. **(B)** FB revealed phlegm clots blocking the bronchus. **(C)** The plastic cast blocking the bronchus was removed. **(D)** The HE staining of lung indicated significant necrosis of the mucosa (200×).

This study has been approved by the ethics committee [Approval number: 2025(028)]. Written informed consent was obtained from at least one guardian of each child in the study. Subjects with RMPP who were hospitalized at the first and third affiliated hospital of Chengdu medical college from January 2023 to May 2025 and met the following diagnostic criteria for RMPP were included: (1) aged < 18 years; (2) clinical respiratory manifestations and signs: including fever, cough or wheezing, with or without abnormal auscultation findings; (3) laboratory tests: positive serological detection of MP immunoglobulin M and positive MP PCR in nasopharyngeal swab samples or bronchoalveolar lavage fluid; (4) imaging tests: chest CT scan showing inflammatory infiltration or consolidation; (5) undergoing flexible bronchoscope (FB); (6) persistent fever (axillary temperature ≥ 38.5 °C), with continuous progression of clinical symptoms and chest imaging signs despite conventional macrolide antibiotic use for 7 days or longer. The diagnostic criteria for RMPP were based on the guidelines for the diagnosis and treatment of RMPP in Children (2023 Edition) (12) and the expert consensus on the diagnosis and treatment of Macrolide-Resistant MPP in Children (13). Based on FB result, the enrolled patients were divided into the PB group and the non-PB group according to whether obstructive mucous casts were formed within the bronchi. Figure 1 showed the discovery in an 8-year-old boy with RMPP under FB (Figure 1).

The exclusion criteria were as follows: (1) patients with chronic lung diseases, pulmonary tuberculosis, primary or secondary immunodeficiency; (2) patients with incomplete clinical data.

### Data collection

Data on general information (sex, age, distribution of age), clinical manifestations (such as duration of fever and cough, chest pain, wheezing, abdominal pain, diarrhea, moist rales, rhonchi, cyanosis) signs, laboratory tests (routine blood tests, inflammatory markers and blood biochemistry), pulmonary imaging (consolidation, bronchiectasis, pleural effusion), FB findings, and treatment were collected at admission.

### Statistical analysis

SPSS software (V23.0, IBM, New York, USA) and R software (V.4.4.2, R Foundation for Statistical Computing, Vienna, Austria) were used for all statistical analyses. Data from both centers were pooled for further analysis. Normally distributed continuous data were presented as means ± standard. Categorical variables were expressed as percentages. The demographic characteristics, clinical manifestation and treatments were compared using Student's t-test, or the chi-square test. Logistic regression analysis was used for correlation analysis. To assess multicollinearity among candidate variables, variance inflation factor was calculated. Based on the results of the previous multivariate analysis, a nomogram was constructed. The discrimination and calibration of the nomogram were evaluated using the area under the receiver operating characteristic curve (AUC), the Hosmer–Lemeshow goodness-of-fit test, and the calibration plot. The decision curve analysis (DCA) was performed to assess the clinical usefulness of the predictive models. A ROC curve was constructed, and bootstrap resampling was repeated 1,000 times to internally verify the nomogram. *P* < 0.05 was considered statistically significant.

## Results

### Demographic characteristics

**Figure 2:**
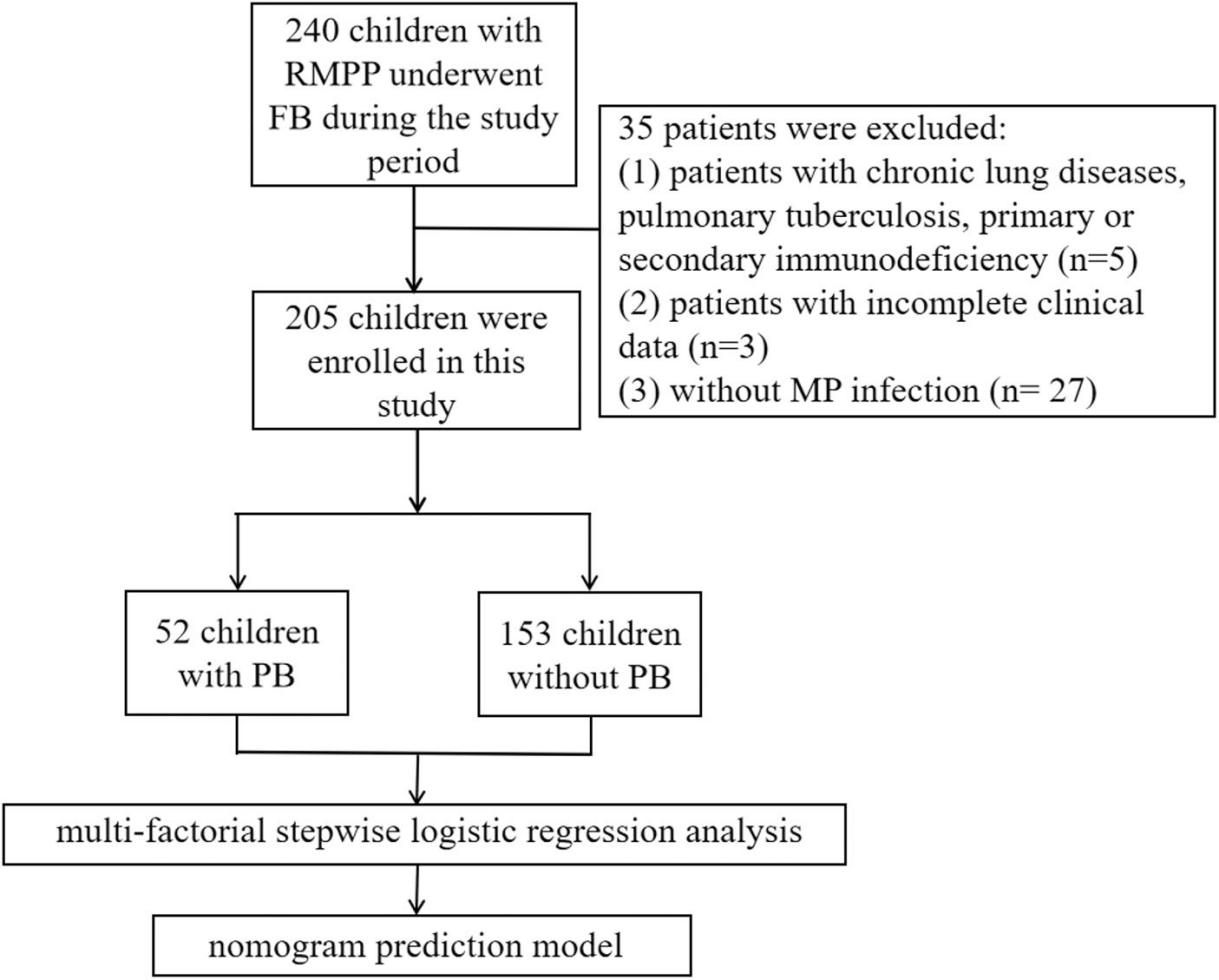
Flowchart of the study population.

A total of 205 children with RMPP met the inclusion criteria, including 52 cases in the PB group and 153 cases in the non-PB group (Figure 2). The average age in the PB group was (7.88 ± 2.61) years, higher than that in the non-PB group, which was (6.89 ± 2.48) years. The age distribution showed that the proportion of children aged 1–5 years in the PB group (21.2%) was lower than that in the non-PB group (37.3%, *P* = 0.033), while the proportion of children aged 10–13 years in the PB group (23.0%) was significantly higher than that in the non-PB group (10.5%, *P* = 0.022). There was no statistically significant difference in gender between the two groups (Table 1).

**Table 1:** The clinical features of patients with or without plastic bronchitis caused by RMPP.

Variables	Non-PB group (*n* = 153)	PB group (*n* = 52)	*P*
Sex (F/M)	74/79	30/22	0.245
Age (mean ± SD,years)	6.89 ± 2.48	7.88 ± 2.61	0.015
Malnutrition [*n*(%)]	7 (4.5)	3 (5.8)	1.000
Distribution of age			
1–5 years [*n*(%)]	57 (37.3)	11 (21.2)	0.033
6–9 years [*n*(%)]	80 (52.3)	29 (55.8)	0.664
10–13 years [*n*(%)]	16 (10.5)	12 (23.0)	0.022
BMI (kg/m2)	16.1 ± 2.3	15.8 ± 2.9	0.442
Duration of fever (days)	3.4 ± 2.7	4.6 ± 2.7	0.005
Peak fever ( °C)			
Low fever (37.3–38.0)	46 (30.1)	8 (15.4)	0.038
Moderate fever (38.1–39.0)	51 (33.3)	10 (19.2)	0.055
High fever (39.1–42.0)	56 (36.6)	34 (65.4)	<0.001
Symptoms			
Cough [*n*(%)]	153 (100)	52 (100)	1.000
Chest pain [*n*(%)]	2 (1.3)	3 (5.8)	0.200
Wheezing [*n*(%)]	2 (1.3)	0 (0)	1.000
Abdominal pain [*n*(%)]	2 (1.3)	0 (0)	1.000
Diarrhea [*n*(%)]	18 (11.8)	1 (1.9)	0.066
Physical signs			
Moist rales [*n*(%)]	106 (69.3)	36 (69.2)	0.995
Rhonchi [*n*(%)]	30 (19.6)	6 (11.5)	0.203
Cyanosis [*n*(%)]	18 (11.8)	8 (15.4)	0.498
Blood testing			
White blood cell (× 10^9^/L)	8.4 ± 3.8	8.0 ± 2.8	0.534
Neutrophil (%)	59.0 ± 12.0	61.9 ± 11.6	0.123
Eosinophils ≥ 3%	14 (1.1)	6 (11.5)	0.616
Hemoglobin(g/L)	129.1 ± 11.8	130.7 ± 12.6	0.612
Platelet (× 10^9^/L)	286.9 ± 111.6	268.1 ± 103.4	0.288
CRP > 20 mg/L	31 (20.3)	24 (46.2)	<0.001
D-dimer ≤ 0.5 mg/L	91 (59.5)	29 (55.8)	0.639
0.5 mg/L < D-dimer ≤ 1.0 mg/L	49 (32.0)	13 (25.0)	0.341
D-dimer > 1.0 mg/L	13 (8.5)	10 (19.2)	0.034
ALT (U/L)	14.3 ± 7.8	14.9 ± 9.0	0.630
AST (U/L)	32.6 ± 7.6	32.9 ± 8.2	0.801
LDH (U/L)	293.7 ± 76.2	310.1 ± 79.5	<0.001
Albumin (g/L)	45.1 ± 2.9	45.1 ± 2.4	0.960
ALP (U/L)	183.5 ± 54.1	174.6 ± 41.6	0.280
ESR (mm/s)	26.4 ± 10.6	30.1 ± 8.8	0.046
Pulmonary imaging			
Consolidation [*n* (%)]	144 (94.1)	50 (96.2)	0.836
Atelectasis [*n* (%)]	13 (8.5)	5 (9.6)	1.000
Bronchiectasis [*n* (%)]	2 (1.3)	0 (0)	1.000
Pleural effusion [*n*(%)]	16 (10.5)	19 (36.5)	<0.001

CRP, C-reactive protein; LDH, lactate dehydrogenase; ALT, alanine transaminase; AST, aspartate transaminase; ALP, alkaline phosphatase; ESR, erythrocyte sedimentation rate.

### Clinical manifestation

The duration of fever in the PB group was longer than that in the non-PB group (4.6 ± 2.7 days vs. 3.4 ± 2.7 days; *P* = 0.005). The proportion of patients with high fever (39.1–42.0 °C) in the PB group was higher than that in the non-PB group (65.4% vs. 36.6%; *P* < 0.001). There were no statistically significant differences between the two groups in the incidence of cough, chest pain, as well as the presence of physical signs such as moist rales, rhonchi, pulmonary consolidation, and atelectasis (Table 1).

### Laboratory tests

There were significant differences in LDH levels [310.1 ± 79.5vs. 293.7 ± 76.2 (U/L), *P* < 0.001]. Compared to non-PB groups, more patients with CRP > 20 mg/L and D-dimer > 1.0 mg/L in PB group

(*P* < 0.05). There was no significant difference in the white blood cell count, neutrophil, eosinophils, hemoglobin, platelet, alanine aminotransferase and aspartate aminotransferase between groups (Table 1).

### Pulmonary imaging

Compared with non-PB group, PB group has higher pleural effusion rate [19 (36.5) vs. 16 (10.5), *P* < 0.001]. There were no differences in the consolidation rate, atelectasis and bronchiectasis between the groups (Table 1).

### Treatment regimens

The distribution of hospitalization time and bronchoscopy frequency was similar between the two groups. Regarding the use of antibiotics and second-line drugs, there was no significant difference in the proportion of macrolide use between the non-PB group and the PB group (*P* > 0.05). However, the proportion of second-line drugs (such as tetracyclines and fluoroquinolones) used in the PB group was showed a significant elevation compared to the in the non-PB group (50.0% vs. 30.7%, *P* = 0.012). Additionally, the proportion of corticosteroid use in the PB group was significantly higher than that in the non-PB group (98.1% vs. 86.3%, *P* = 0.018) (Table 2).

**Table 2:** The treatment of patients with or without plastic bronchitis caused by RMPP.

Variables	Non-PB group (*n* = 153)	PB group (*n* = 52)	*P*
Hospital stay (days)			
1–7 [*n* (%)]	21 (13.7)	7 (13.5)	0.962
8–14 [*n* (%)]	128 (83.7)	44 (84.6)	0.871
>14 [*n* (%)]	4 (2.6)	1 (1.9)	1.000
Bronchoscopy frequency			
1 [*n* (%)]	148 (97.3)	49 (94.2)	0.696
2 [*n* (%)]	5 (2.7)	3 (5.8)	0.696
Therapy			
Macrolides [*n* (%)]	65 (42.5)	18 (34.6)	0.318
Second-line drugs [*n* (%)]	47 (30.7)	26 (50.0)	0.012
Corticosteroids [*n* (%)]	132 (86.3)	51(98.1)	0.018

### Multivariate regression analysis of PB in patients with RMPP

Variables with a *P*-value < 0.1 in the univariate analysis (age, duration of fever, peak fever, CRP > 20 mg/L, LDH, D-dimer > 1.0 mg/L, ESR, pleural effusion, and diarrhea) were entered into the multivariate logistic regression model. According to the collinearity diagnosis, the tolerance and the variance inflation factor of variables in the logistic regression model were >0.1 and <10, respectively. Furthermore, according to the principle of collinearity diagnosis (14), no collinearity among the independent variables was observed in this study (Table 3).

**Table 3:** Collinear diagnosis of the independent variables (*n* = 205).

Variables	Tolerance	Variance inflation factor
Age	0.869	1.150
Duration of fever	0.737	1.357
Peak fever	0.724	1.381
CRP > 20 mg/L	0.842	1.187
LDH	0.690	1.449
D-dimer > 1.0 mg/L	0.836	1.197
ESR	0.865	1.156
Pleural effusion	0.909	1.100
Diarrhea	0.811	1.233

Finally, multivariate stepwise logistic regression analysis identified CRP > 20 mg/L (OR = 3.389, 95% CI: 1.474–7.796), pleural effusion (OR = 4.571, 95% CI: 1.751–11.933), and LDH (per unit increase: OR = 1.005, 95% CI: 1.001–1.010; per 10 U/L increase: OR = 1.051, 95% CI: 1.011–1.093) as independent risk factors for the development of PB in patients with RMPP (Table 4).

**Table 4:** Multi-factorial stepwise logistic regression analysis.

Variables	*β*	S.E.	*P*	OR	95%CI
CRP>20 mg/L	1.221	0.425	0.004	3.389	1.474–7.796
Pleural effusion	1.52	0.49	0.002	4.571	1.751–11.933
LDH	0.005	0.002	0.030	1.005	1.001∼1.010
Constant	−4.365	1.088	<0.001	0.013	0.002- 0.107

### Nomogram prediction model of PB in patients with RMPP

**Figure 3:**
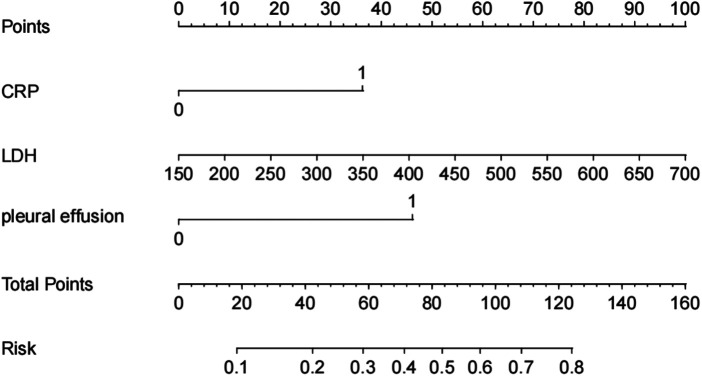
Nomogram of regression equations for calculating the risk score and predicting the risk of PB in children with RMPP. The variables with *P* < 0.1 in univariate analysis were included in the multivariate regression model for regression analysis. Finally, multivariate stepwise regression analysis was conducted to examine the risk factors for PB.

**Figure 4:**
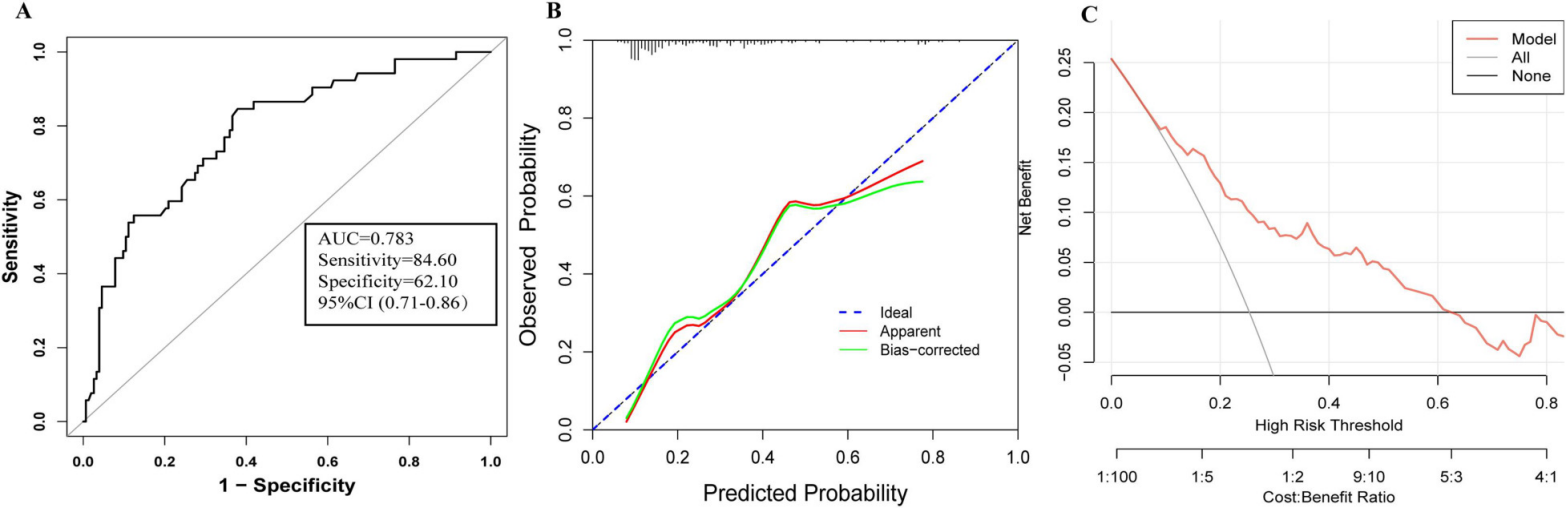
ROC, calibration, and DCA evaluating the predictive performance of PB in children with *Mycoplasma pneumoniae* pneumonia. **(A)** ROC curve analysis for the nomogram model. **(B)** Calibration curves comparing predicted risk (horizontal axis) and observed outcomes (vertical axis). Predictors included pleural effusion, CRP >20 mg/L, and LDH levels (enclosed in brackets). **(C)** DCA assessing clinical utility of the model. The straight line represents the assumption that all patients develop PB; the horizontal line denotes the assumption that no patient will develop PB.

A nomogram for predicting the risk of PB was constructed based on the three independent risk factors identified by logistic regression analysis (Figure 3). The ROC curve for the nomogram was 0.783, with a cut-off value of 0.172 yielding a sensitivity of 84.6% and a specificity of 62.1% (Figure 4A). Higher total scores derived from the nomogram, calculated by summing the assigned points for each risk factor, were associated with an increased probability of PB. The Hosmer–Lemeshow goodness-of-fit test yielded a nonsignificant result (*P* = 0.408, R^2^ = 8.269), suggesting adequate model calibration. Calibration plots demonstrated good agreement between predicted probabilities and observed outcomes (Figure 4B). Decision curve analysis further confirmed the clinical utility of the model (Brier score=0.154, Emax=0.136; Figure 4C).

## Discussion

RMPP is typically characterized by a long course of disease, poor treatment outcomes, and numerous complications, which can be life-threatening in severe patients. Recently, the incidence of RMPP has increased by as much as 40%, highlighting the growing importance of early detection and management of complications (4). PB, recognized as one of the serious complications of RMPP, presents with a gradual onset and a significantly high mortality rate. Therefore, our study aims to develop a nomogram prediction model for the occurrence of PB in RMPP based on clinical data, facilitating early intervention and providing clinical guidance.

Common clinical manifestations of RMPP include cough, persistent fever, and chest pain, which are consistent with the symptoms observed in children with RMPP complicated with PB in this study. Our further analysis indicates that both the peak temperature and duration of fever in the PB group are higher than those in the non-PB group, suggesting a more severe inflammatory response in the PB group (4, 8), Additionally, both local immune responses and systemic immune-inflammatory reactions in the PB group are stronger than those in the non-PB group (3). Furthermore, ZHANG et al. have identified that in children with RMPP experiencing high fever, the accelerated evaporation of bodily fluids can lead to moisture loss in the respiratory tract, resulting in thickened mucus secretions in the trachea and fostering the development of PB (4). Moreover, we found that the average age of children in the PB group is higher than that in the non-PB group [(7.88 ± 2.61) vs. (6.89 ± 2.48) years], mainly occurring in preschool children. The research conducted by Wang Z et al. aligns closely with our findings, suggesting that age is a critical factor influencing the prognosis of RMPP (3). The reason may be potentially linked to the underdeveloped immune responses in preschoolers. A robust cellular immune response may lead to necrosis of airway epithelial cells and significant ciliary dysfunction in children with RMPP, thereby impairing airway immune function and mucociliary clearance, which increases the likelihood of mucus plug formation (6, 8).

Our study suggested that levels of CRP, LDH, and ESR in the PB group are significantly higher than those in the non-PB group (*P* < 0.05). Multivariate regression analysis identifies CRP > 20 mg/L, pleural effusion, and elevated LDH as independent risk factors for PB in children with RMPP. CRP levels serve as effective markers for monitoring disease progression, assessing prognosis, and reflecting the severity of the inflammatory response linked to MP infection. Gong et al. identified CRP > 40 mg/L as a risk factor for RMPP (7, 9), while Zhang et al. indicated that CRP ≥ 16.5 mg/L is a risk factor (10). These cut-off values are different from the results of our study. This difference may be related to factors such as bacterial infection in the research subjects, early intervention to inhibit the inflammatory response, and detection timing. In the early phases of RMPP infection, CRP levels may not peak. Therefore, dynamic monitoring of CRP is crucial for the early identification of PB. An ongoing rise in CRP necessitates alert regarding the potential onset of PB and prompt intervention with FB. Additionally, LDH, an enzyme that facilitates the conversion of pyruvate to lactic acid, is released into the serum following cellular damage and is frequently utilized as a key indicator for inflammatory diseases in clinical practice (10). For PB in children with RMPP, an excessive pulmonary inflammatory response increases cell membrane permeability, leading to augmented release of intracellular LDH into the bloodstream (15). Some recent study found elevated LDH levels as critical predictors of RMPP occurrence (11–13), and our study further found that increased LDH is also an independent risk factor for PB in children with RMPP. In addition, the increase in LDH activity is related to the severity of pulmonary inflammation, and LDH can be used as a marker for glucocorticoid therapy in RMPP (8), and high LDH levels often indicate poor response to glucocorticoid therapy in RMPP children (16).

Imaging features provide an important basis for clinical judgment of pulmonary conditions and complications in RMPP children. Chest imaging findings in RMPP children often show inflammatory infiltration, atelectasis, pleural effusion, and may progress to bronchiolitis obliterans (17). Our findings showed that pleural effusion is an independent risk factor for PB in RMPP children, aligning with results from various studies (18, 19). The reasons why RMPP complicated with PB is prone to pleural effusion may be as follows: RMPP children have severe systemic inflammatory responses, and cytokine storms promote neutrophil infiltration, aggravating the pleural reaction and leading to significant exudation and pleural effusion. In children in the PB group, airway obstruction due to mucus plugging results in more intense pleural responses, increasing the likelihood of pleural effusion (20).

We established a nomogram prediction model based on three indicators, namely CRP > 20 mg/L, pleural effusion, and elevated LDH. This model demonstrated an AUC ROC of 0.783 (95% CI 0.71–0.86), indicating strong reliability. Zhang et al. constructed a nomogram model based on 5 risk factors (persistent fever before FB, extra-pulmonary complications, pleural effusion, duration of cough, and LDH level) for early identification of PB in RMPP children (21). Shen et al.'s study showed that the RMPP nomogram prediction model constructed by fever duration exceeding 10.5 days, pleural effusion, white blood cells > 10.13 × 10^9^/L, neutrophil count > 6.43 × 10^9^/L, CRP > 29.45 mg/L, LDH > 370.50 U/L, neutrophil-to-lymphocyte ratio > 3.47, and serum uric acid < 170.5 μmol/mL has good predictive performance (16). The above studies all indicate that inflammatory factors (CRP, LDH) combined with imaging examinations (pleural effusion) can effectively predict early identification of PB caused by RMPP.

This study did not investigate the optimal timing of bronchoscopic intervention. Previous studies have indicated that bronchoscopy in children with PB should be performed earlier than in those without PB, particularly within 4–10 days of disease onset, to achieve satisfactory clinical outcomes (22). Furthermore, evidence suggests that even earlier intervention (e.g., within 12 h of admission) may reduce the risk of dyspnea in children with PB caused by Mycoplasma pneumoniae infection (23). In our study, the PB group exhibited elevated inflammatory markers—CRP >20 mg/L, pleural effusion, and increased LDH levels—indicating a pronounced systemic inflammatory response. Although no significant difference was observed in the incidence of secondary fiberoptic bronchoscopy (FB) between the PB and non-PB groups, previous studies have demonstrated that cast removal via bronchoscopy rapidly alleviates respiratory obstruction and inflammation, potentially reducing the need for repeat FB (24). The lack of a significant difference in our study may be attributable to the limited sample size. Notably, substantial heterogeneity exists across studies regarding the indications and timing of bronchoscopic intervention, which may be influenced not only by variations in disease progression and clinical characteristics but also by clinicians’ awareness and experience with PB.The optimal clinical threshold for recommending bronchoscopy warrants further investigation.

This study has several limitations: (1) it is a retrospective analysis conducted at two centers, and (2) the sample size is limited, lacking prospective cohorts to validate the nomogram model. And the reliability of the predictive indicators warrants further validation through larger, multi-center, prospective studies.

## Conclusion

The incidence of PB in children with RMPP is 25.4%. We established a nomogram prediction model based on three indicators, namely CRP > 20 mg/L, pleural effusion, and elevated LDH. This nomogram may facilitate early screening of high-risk children in clinical practice, potentially reducing adverse outcomes.

## Data Availability

The raw data supporting the conclusions of this article will be made available by the authors, without undue reservation.

## References

[1] Li Y Williams RJ Dombrowski ND Watters K Daly KP Irace AL Current evaluation and management of plastic bronchitis in the pediatric population. Int J Pediatr Otorhinolaryngol. (2020) 130:109799. 10.1016/j.ijporl.2019.109799 31812839 PMC9187852

[2] Huang F Gu W Diwu J Zhang X He Y Zhang Y Etiology and clinical features of infection-associated plastic bronchitis in children. BMC Infect Dis. (2023) 23(1):588. 10.1186/s12879-023-08529-w 37679703 PMC10486060

[3] Tamura A Matsubara K Tanaka T Nigami H Yura K Fukaya T. Methylprednisolone pulse therapy for refractory Mycoplasma pneumoniae pneumonia in children. J Infect. (2008) 57(3):223–8. 10.1016/j.jinf.2008.06.012 18656264 PMC7112643

[4] Huang W Xu X Zhao W Cheng Q. Refractory Mycoplasma pneumonia in children: a systematic review and meta-analysis of laboratory features and predictors. J Immunol Res. (2022) 2022:9227838. 10.1155/2022/9227838 35795531 PMC9251082

[5] Yang L Zhang Y Shen C Lu Z Hou T Niu F Clinical features and risk factors of plastic bronchitis caused by Mycoplasma pneumoniae pneumonia in children. BMC Pulm Med. (2023) 23(1):468. 10.1186/s12890-023-02766-0 37996853 PMC10668422

[6] Huang JJ Yang XQ Zhuo ZQ Yuan L. Clinical characteristics of plastic bronchitis in children: a retrospective analysis of 43 cases. Respir Res. (2022) 23(1):51. 10.1186/s12931-022-01975-1 35248022 PMC8898471

[7] Walker PA Shah SK Letourneau PA Allison ND Cox CS. Treatment of plastic bronchitis using serial flexible bronchoscopy and aerosolized heparin therapy. Eur J Pediatr Surg. (2013) 23(2):157–60. 10.1055/s-0032-1315803 22782325

[8] Zhong H Yin R Zhao R Jiang K Sun C Dong X. Analysis of clinical characteristics and risk factors of plastic bronchitis in children with Mycoplasma pneumoniae pneumonia. Front Pediatr. (2021) 9:735093. 10.3389/fped.2021.735093 34733807 PMC8558491

[9] Tian XY Zhang GL Wang CJ Gu RX Li YY Li QY [Clinical characteristics of plastic bronchitis and risk factors for recurrence in children]. Zhongguo Dang Dai Er Ke Za Zhi. (2023) 25(6):626–32. 10.7499/j.issn.1008-8830.2211122 37382133 PMC10321426

[10] Zhan XW Deng LP Wang ZY Zhang J Wang MZ Li SJ. Correlation between Mycoplasma pneumoniae drug resistance and clinical characteristics in bronchoalveolar lavage fluid of children with refractory Mycoplasma pneumoniae pneumonia. Ital J Pediatr. (2022) 48(1):190. 10.1186/s13052-022-01376-6 36435821 PMC9701416

[11] Zhao L Zhang T Cui X Zhao L Zheng J Ning J Development and validation of a nomogram to predict plastic bronchitis in children with refractory Mycoplasma pneumoniae pneumonia. BMC Pulm Med. (2022) 22(1):253. 10.1186/s12890-022-02047-2 35761218 PMC9235233

[12] China. NHCotPsRo. Guidelines for diagnosis and treatment of Mycoplasma pneumonia in children (2023 edition). J Emerg Infect Dis. (2024) 9(1):73–9. 10.19871/j.cnki.xfcrbzz.2024.01.015

[13] Wang YS Zhou YL Bai GN Li SX Xu D Chen LN Expert consensus on the diagnosis and treatment of macrolide-resistant Mycoplasma pneumoniae pneumonia in children. World J Pediatr. (2024) 20(9):901–14. 10.1007/s12519-024-00831-0 39143259 PMC11422262

[14] Cheng J Sun J Yao K Xu M Cao Y. A variable selection method based on mutual information and variance inflation factor. Spectrochim Acta A Mol Biomol Spectrosc. (2022) 268:120652. 10.1016/j.saa.2021.120652 34896682

[15] Tong L Huang S Zheng C Zhang Y Chen Z. Refractory Mycoplasma pneumoniae pneumonia in children. early recognition and management. J Clin Med. (2022) 11(10):2824. 10.3390/jcm11102824 35628949 PMC9144103

[16] Shen W Sun X. Construction of a nomogram for early diagnosis of refractory Mycoplasma pneumoniae pneumonia in children. Transl Pediatr. (2024) 13(7):1119–29. 10.21037/tp-24-16 39144443 PMC11320014

[17] Cheng Q Zhang H Shang Y Zhao Y Zhang Y Zhuang D Clinical features and risk factors analysis of bronchitis obliterans due to refractory Mycoplasma pneumoniae pneumonia in children: a nomogram prediction model. BMC Infect Dis. (2021) 21(1):1085. 10.1186/s12879-021-06783-4 34674642 PMC8529771

[18] Choi YJ Chung EH Lee E Kim CH Lee YJ Kim HB Clinical characteristics of macrolide-refractory Mycoplasma pneumoniae pneumonia in Korean children: a multicenter retrospective study. J Clin Med. (2022) 11(2):306. 10.3390/jcm11020306 35054002 PMC8779611

[19] Zhou Y Wang J Chen W Shen N Tao Y Zhao R Impact of viral coinfection and macrolide-resistant mycoplasma infection in children with refractory Mycoplasma pneumoniae pneumonia. BMC Infect Dis. (2020) 20(1):633. 10.1186/s12879-020-05356-1 32847534 PMC7447613

[20] Wang M Wang Y Yan Y Zhu C Huang L Shao X Clinical and laboratory profiles of refractory Mycoplasma pneumoniae pneumonia in children. Int J Infect Dis. (2014) 29:18–23. 10.1016/j.ijid.2014.07.020 25449230

[21] Zhang H Yang J Zhao W Zhou J He S Shang Y Clinical features and risk factors of plastic bronchitis caused by refractory Mycoplasma pneumoniae pneumonia in children: a practical nomogram prediction model. Eur J Pediatr. (2023) 182(3):1239–49. 10.1007/s00431-022-04761-9 36633659 PMC10023623

[22] Huang JJ Yuan L Zhuo ZQ Zhu QG Li MZ. Clinical analysis of plastic bronchitis caused by adenoviral pneumonia in 9 children. Chin J Appl Clin Pediatr. (2020) 35(16):1260–3. 10.3760/cma.j.cn101070-20190802-00706

[23] Wang L Wang W Sun JM Ni SW Ding JL Zhu YL Efficacy of fiberoptic bronchoscopy and bronchoalveolar lavage in childhood-onset, complicated plastic bronchitis. Pediatr Pulmonol. (2020) 55(11):3088–95. 10.1002/ppul.25016 32770770

[24] Wang Y An S. Plastic bronchitis associated with influenza A virus in children with asthma. J Int Med Res. (2021) 49(12):3000605211065370. 10.1177/03000605211065370 34939439 PMC8721730

